# Telocytes in liver: electron microscopic and immunofluorescent evidence

**DOI:** 10.1111/jcmm.12195

**Published:** 2013-11-25

**Authors:** Junjie Xiao, Fei Wang, Zhenguo Liu, Changqing Yang

**Affiliations:** a Regeneration Lab and Experimental Center of Life Sciences, School of Life Science, Shanghai UniversityShanghai, China; b Innovative Drug Research Center, Shanghai UniversityShanghai, China; c Division of Gastroenterology and Hepatology, Digestive Disease Institute, Tongji Hospital, Tongji University School of MedicineShanghai, China

**Keywords:** telocytes, telopodes, liver, CD34, c-kit, vimentin, PDGFR-α, β, hepatic putative stem cells

## Abstract

Hepatic interstitial cells play a vital role in regulating essential biological processes of the liver. Telocytes (TCs), a novel type of interstitial cells firstly identified by Popescu and his coworkers, have been reported in many tissues and organs, but not yet in liver (go to http://www.telocytes.com). We used transmission electron microscopy and immunofluorescence (double labelling for CD34 and c-kit/CD117, or vimentin, or PDGF Receptor-α, or β) to provide evidence for the existence of TCs in mice liver. The distribution of TCs in liver was found to be of similar density in the four hepatic lobes. In conclusion, here we show the presence of TCs in mice liver. It remains to be determined the possible roles of TCs in the control of liver homeostasis and regeneration, the more so as a close special relationship was found between TCs and hepatic putative stem (progenitor) cells.

## Introduction

Telocytes (TCs) are a novel type of stromal cells of mesenchymal origin firstly identified by Popescu’s group 1–14 and adopted by other laboratories worldwide (go to http://www.telocytes.com) 15–26. The most impressing feature of TCs is their extremely long prolongations telopodes (Tps) extending from the cell body. Telopodes present dilatations called podoms and thin segments (below the resolving power of light microscopy, named podomeres) 2–8. Telocytes are connected by Tps and consequently form a network 24–27. The microRNA signatures 28 of TCs as well as the gene profiles 29 were established. The electrophysiological characteristics of isolated TCs were described 13. As a distinct population of interstitial cells, TCs have been documented in the interstitial space of many organs and tissues in mammalian 2–15. These organs include not only cavitary ones but also non-cavitary organs like pancreas 30. The cavitary organs include heart, intestine, uterus, pulmonary veins *etc*. while the non-cavitary organs include skeletal muscle, pancreas, placenta, mammary gland, *etc*. 2–30. Thus, it is reasonable to hypothesize that TCs may exist in almost all organs 30. However, it remains to be determined that TCs are present in the liver.

This study was aimed to investigate the existence of TCs in the liver by transmission electron microscopy (TEM) as this technique assures the precise identification of TCs 30. In addition, we used immunofluorescence methods, particularly the double labelling for CD34 and PDGFR-α considered at present as the immunohistochemical marker for TCs in gastrointestinal tract 31.

## Materials and methods

### Animals

C57/BL6 male mice (25–30 g) aged 10–12 weeks, purchased from Shanghai SLAC Laboratory Animal CO. LTD (Shanghai, China) were used in this study. The mice were housed in a temperature-controlled facility with a 12 hr light/dark cycle with full access to water and food for at least 1 week for the experiment. This study was approved by the local ethical committees and all animal experiments were conducted under the guidelines on humane use and care of laboratory animals for biomedical research published by National Institutes of Health (No. 85-23, revised 1996).

### Transmission electron microscopy

Tissues were cut into 1 mm^3^ fragments and fixed by immersion in 5% glutaraldehyde in phosphate buffer (0.1 M, pH 7.4) overnight at 4°C. After that, it was washed in phosphate buffer for four times followed by post-fixation with 1% osmium tetroxide in 0.1 M phosphate buffer for 2 hrs at 4°C. Tissues were dehydrated through graded alcohols (50, 70, 90 and 100%) for 30 min. each and embedded in Epon 812. Semithin sections were cut at 1.5 μm and stained with toluidine blue, and histologically analysed by light microscopy. Ultrathin sections were cut at 70 nm and contrasted with uranyl acetate and lead citrate, and they were examined with a JEM-1010 electron microscope (JEOL, Tokyo, Japan). Snap-shots were taken using a video camera Veleta and the iTEM Olympus Soft Imaging System (Tokyo, Japan).

### Immunofluorescent staining

Frozen sections (6 μm thick) were mounted on Superfrost Plus slides (Shitai, China). Sections were fixed in paraformaldehyde for 15 min. After washed with PBS for three times, sections were pre-incubated in PBS supplemented with 10% goat serum for 1 hr, and then incubated overnight at 4°C with rabbit polyclonal anti-c-kit (ab5506; Abcam, Cambridge, UK) and rat monoclonal anti-CD34 (ab8158; Abcam). Both antibodies were diluted by 1:100 in 1× PBS with 0.25% Triton X-100. After that, sections were exposed for 1 hr to goat anti-rat labelled with FITC (sc-2011; Santa Cruz, Dallas, TX, USA) and goat anti-rabbit labelled with rhodamine secondary antibodies (sc-362262; Santa Cruz) diluted by 1:200 in the same buffer. Finally, sections were stained with 4′,6-diamidino-2-phenylindole (DAPI) (ProLong® Gold; Life technology, Carlsbad, CA, USA). The same protocol was used in Rabbit polyclonal to PDGF Receptor-alpha (ab61219, 1:100; Abcam), Rabbit monoclonal to PDGF Receptor-beta (ab32570, 1:100; Abcam) and Rabbit monoclonal to Vimentin (ab92547, 1:100; Abcam).

### Semi-quantification of hepatic TCs

Representative sections of the left lateral, right, median and caudate lobes of mice livers were used for immuofluorescent staining. For the semi-quantification of hepatic TCs, double staining for c-kit and CD34 was used. Each lobe of the liver was randomly obtained of 20 images (400×) in the central area using confocal laser scanning microscope (LSM 710; Carl Zeiss MicroImaging GmbH, Jena, Germany). The anti-c-kit and CD34 images from the same field were merged by Zen 2011 software (Carl Zeiss MicroImaging GmbH). Three mice were used in this experiment. Counting of the hepatic TCs was performed in a double-blinded method. The density of TCs was expressed as TCs number/number of DAPI-stained nuclei.

### Statistical analysis

Data were presented as mean ± SD. A one-way anova was conducted to evaluate the one-way layout data. If a significant difference was observed, Bonferroni’s *post hoc* test was conducted to identify groups with significant differences. All analyses were performed with SPSS 17.0, (IBM SPSS Statistics, Armonk, NY, USA) and all statistical tests were two-sided. *P*-values that were less than 0.05 were considered to be statistically significant.

## Results

**Figure 1:**
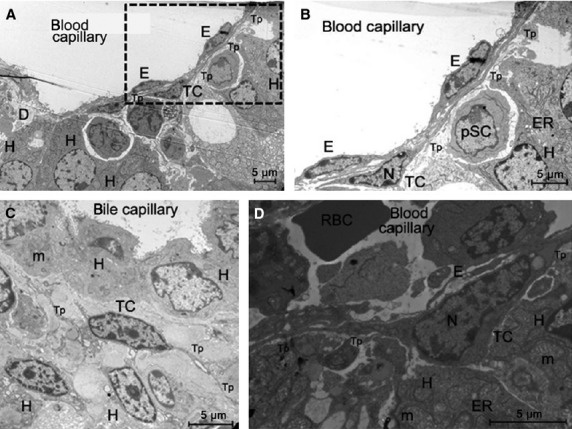
Electron microscope images showing the ultrastructure of liver (mice). (A) Telocytes (TCs) with telopodes (Tps) in the Disse space (D) between endothelial cells (E) and hepatocytes (H). Note the upper telopode (Tp) which is more than 20 μm long. (B) Higher magnification of the field inside the rectangle in A. Note in between the TC and hepatocytes (H) the presence of a putative stem cell (pSC) which has the features of a young cell (progenitor cell?); ER, endoplasmic reticulum; N, nucleus. (C) A TC with at least three Tps; H, hepatocyte; m, mitochondria. (D) A TC with a heterochromatic nucleus (N) at a higher magnification; E, endothelial cell; RBC, red blood cell; H, hepatocyte; m, mitochondria; Tp, telopodes; scale bar = 5 μM.

Transmission electron microscopy examination is a golden standard for the identification of TCs 1–30. As shown in Figure 1, TCs were present in the Disse space of the liver and had the distinctive ultrastructural features. Observed in other organs 1–19 TCs have oval or triangular-shaped cell body, containing a slightly heterochromatic nucleus. Telocytes present 2–3 Tps with the characteristic conformations: alternation of thin long portions (podomers) and dilated segments (podoms).

**Figure 2:**
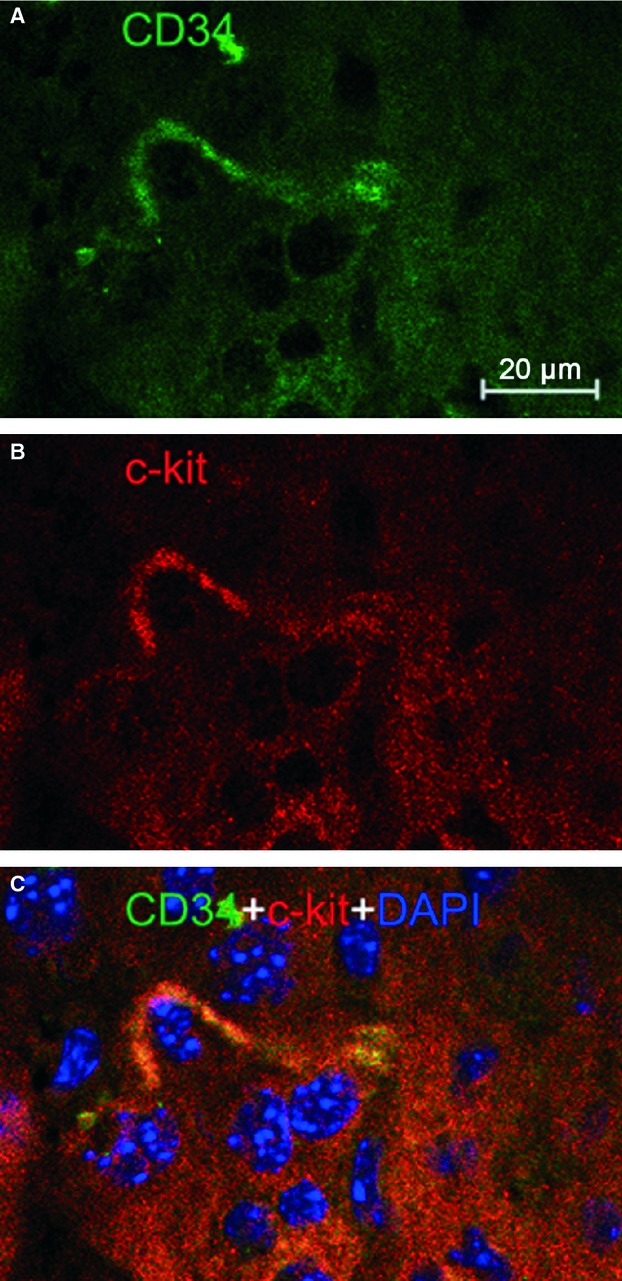
C-kit/CD34 double immunofluorescence labelling shows a telocyte. Laser scanning confocol microscopy: double immunofluorescence labelling shows (A) CD34 (green), (B) c-kit (red) and (C) co-localization (yellow) in a telocyte. Nuclei are counterstained with DAPI (blue). Original magnification 400 ×; scale bar = 20 μm.

**Figure 3:**
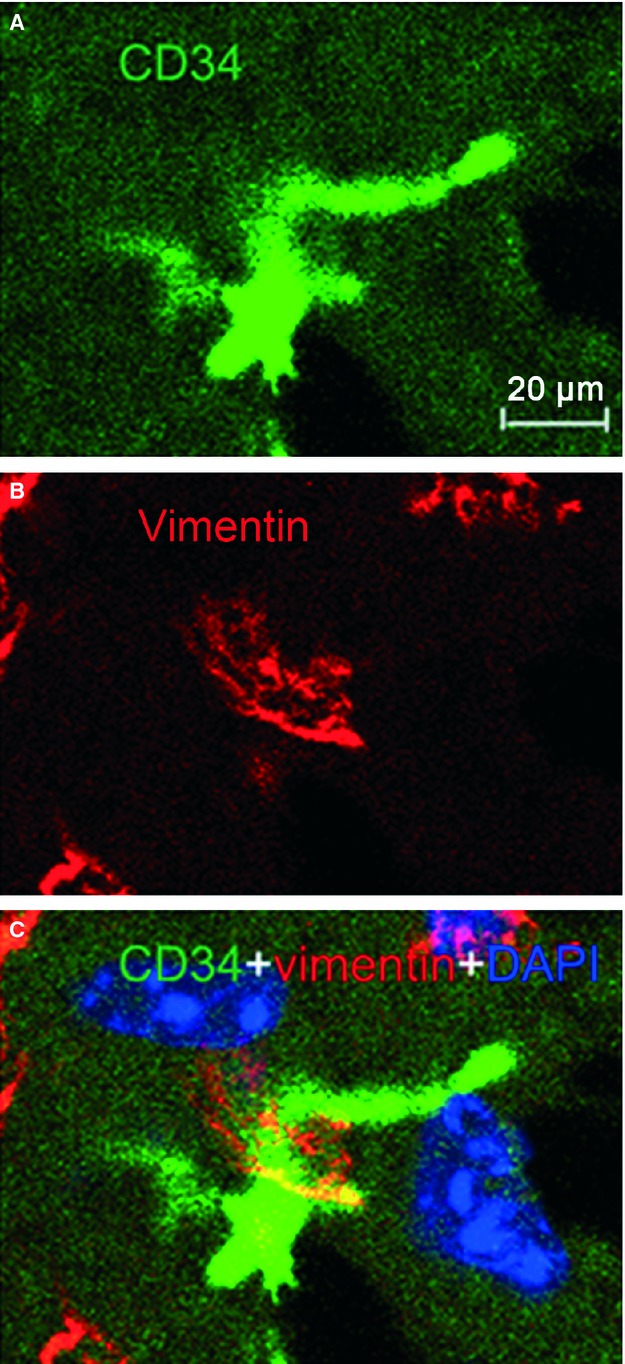
Vimentin/CD34 double immunofluorescence labelling shows a telocyte. Laser scanning confocol microscopy: double immunofluorescence labelling shows (A) CD34 (green), (B) vimentin (red) and (C) co-localization (yellow) in a telocyte. Nuclei are counterstained with DAPI (blue). Original magnification 400 ×; scale bar = 20 μm.

**Figure 4:**
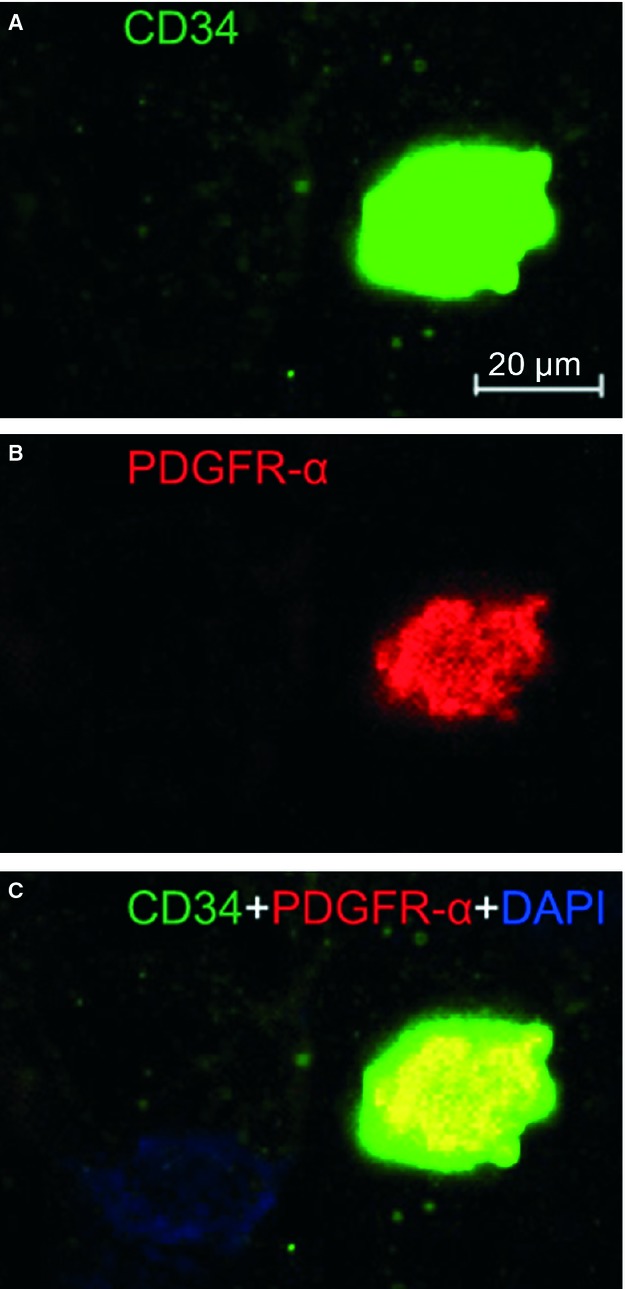
PDGFR-α/CD34 double immunofluorescence labelling shows a telocyte. Laser scanning confocol microscopy: double immunofluorescence labelling shows (A) CD34 (green), (B) PDGFR-α (red) and (C) co-localization (yellow) in a telocyte. Nuclei are counterstained with DAPI (blue). Original magnification 400 ×; scale bar = 20 μm.

**Figure 5:**
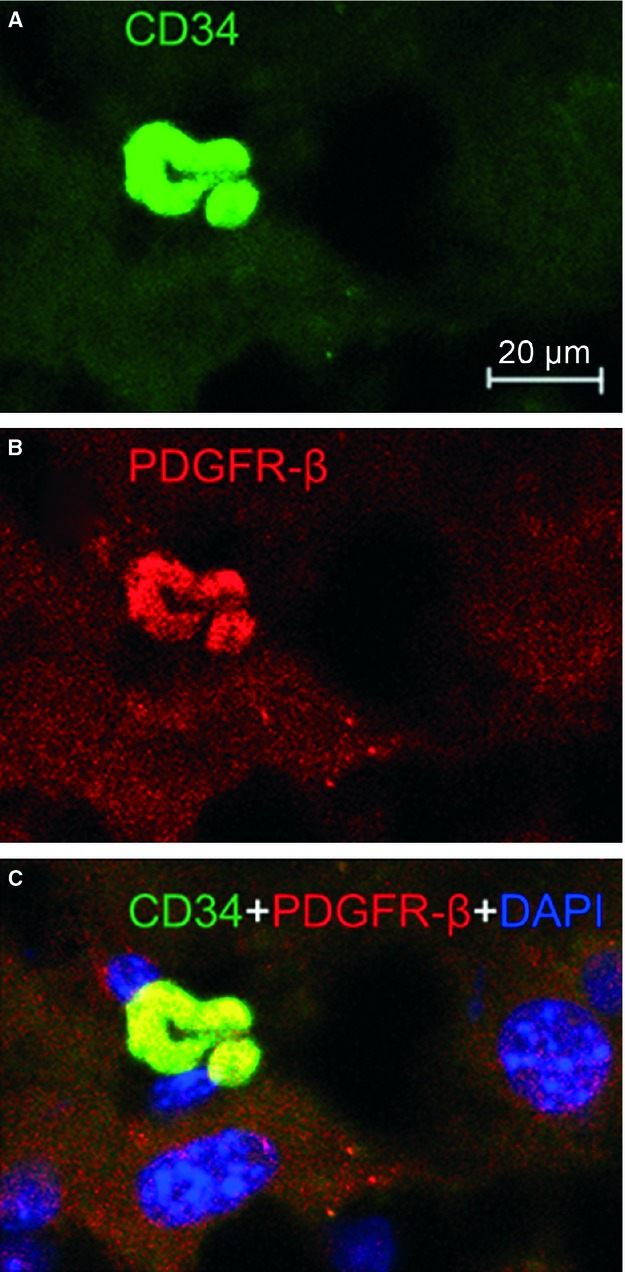
PDGFR-β/CD34 double immunofluorescence labelling shows a telocyte. Laser scanning confocol microscopy: double immunofluorescence labelling shows (A) CD34 (green), (B) PDGFR-β (red) and (C) co-localization (yellow) in a telocyte. Nuclei are counterstained with DAPI (blue). Original magnification 400 ×; scale bar = 20 μm.

Immunofluorescence is also of importance to determine the phenotype of TCs 1–33. Four different double labelling immunofluorescence methods were used to provide evidence for the existence of TCs in mice liver. These methods include double labelling for CD34 and c-kit/CD117, CD34 and vimentin, CD34 and PDGFR-α, and CD34 and PDGFR-β 30–31. Figure 2 showed CD34/c-kit double-positive cells whereas Figure 3 presented CD34/vimentin double-positive cells. Similarly, Figures 4 and 5 identified CD34/PDGFR-α double-positive cells and CD34/PDGFR-β double-positive cells, respectively. Figure 5 show the positive results of the double labelling. Thus, four different double staining methods provided evidence for the existence of TCs in the liver.

A similar distribution of TCs was found in the four hepatic lobes, when expressed as TCs number/number of DAPI-stained nuclei: namely, as percentage: 1.7 ± 0.6% left lateral lobe; 1.8 ± 0.5% median lobe; 1.9 ± 0.5% right lobe; 1.8 ± 0.6% caudate lobe (*P* = 0.823). Therefore, no significant difference appeared.

## Discussion

This study shows TCs as a distinct population of cells, distinguished from other interstitial cells (mainly Kupffer cells and hepatic stellate cells) in liver by their location, morphology and immunophenotypes. Kupffer cells are located inside the sinusoids 32 while the cells identified in the present study are in the space of Disse. Hepatic stellate cells, also known as Ito cells are pericytes found in the space of Disse 32. Although the cells described in this study are also located in the space of Disse, they have characteristic very long prolongations (Tps) and specific biomarkers (double-positive for CD34 and c-kit/CD117, or vimentin, or PDGF-α, or PDGF–β), making them different from hepatic stellate cells in both morphology and immunophenotype 30.

The precise functions of TCs in liver remain to be established. However, based on the literature, at least three relevant and potential roles could be proposed: (*i*) intercellular connections *via* Tps 1–30, (*ii*) intercellular signalling *via* shedding vesicles or paracrine secretion 1–30 and (*iii*) liver regeneration as was supported for heart 9–33. It is highly needed to explore the potential functions of TCs in the pathological conditions of the liver and the interaction between TCs and other cells. Further studies are required to investigate the role of TCs in liver fibrosis as reported in systemic sclerosis 24.

In conclusion, this study firstly demonstrated the presence of TCs in liver based on the specific ultrastructural and immunofluorescent characteristics. The presence of TCs in the liver opens a new window for better understanding responses that have not been determined in hepatic biology. Telocytes may be a new kind of target cells for the treatment and prevention of liver diseases.
